# Burden tests can be used to map causal genes for a simple metabolic trait in an exome‐sequenced polyploid mutant population

**DOI:** 10.1111/pbi.13890

**Published:** 2022-07-25

**Authors:** Guillaume N. Menard, Peter J. Eastmond

**Affiliations:** ^1^ Plant Sciences and the Bioeconomy Rothamsted Research Hertfordshire UK

**Keywords:** burden test, rare‐variant association analysis, mutant population, wheat

Forward genetic screens are an excellent tool to assign gene function, but it is often necessary to employ map‐based cloning to identify the causal genes. This can be laborious and represents a bottleneck in plant fundamental and applied research. With advances in DNA technology, it is becoming increasingly affordable to sequence large populations. Krasileva *et al*. (2017) exome sequenced tetraploid and hexaploid wheat ethyl methanesulfonate (EMS) mutagenized populations, primarily to facilitate reverse genetic screens. Gene redundancy allows a very high mutant load of 35–40 mutations per kilobase, and the populations of ~1500 and ~1200 lines each harbour ~22–23 missense or truncation mutations per gene. Here, we show that burden tests, a simple form of rare‐variant association analysis developed for human disease genetics (Lee *et al*., 2014), can be used to identify causal genes in the hexaploid wheat (*Triticum aestivum*) cv. Cadenza mutant population, without the need for map‐based cloning.

The statistical power to detect association with rare variants is very limited (Lee *et al*., 2014), and most mutations in the Cadenza EMS population are singletons (Krasileva *et al*., 2017). Burden tests work by collapsing multiple variants within a gene (or other functional groups) into a single test score, thereby increasing frequency and providing greater power (Lee *et al*., 2014). However, this power relies on the selected variants mostly being causal and having the same direction and magnitude of effect (Lee *et al*., 2014). Such assumptions likely hold for mutant populations where causal variants are most frequently deleterious (Meinke, 2013), and their severity can be predicted from sequence analysis (Kumar *et al*., 2009). The absence of genetic structure in mutant populations should simplify association studies and collapsing homoeologous groups, that lack functional divergence in ‘recent’ polyploids like wheat (Krasileva *et al*., 2017), should also improve power.

To investigate whether burden tests can be applied to the Cadenza population, we measured the fatty acid composition of lipids in individual M_4_ grains (caryopses) from 1188 exome‐sequenced lines using gas chromatography and calculated the proportion of unsaturated fatty acids that are polyunsaturated (ω‐6 desaturation efficiency or ω‐6DE), which is a simple adaptive metabolic trait (Menard *et al*., 2017) and a determinant of edible oil quality (Hajiahmadi *et al*., 2020). As summarized in Figure 1a, we extracted a list of putative deleterious mutations in the M_2_ population (Krasileva *et al*., 2017) using BioMart within EnsemblPlants (https://plants.ensembl.org/biomart/martview) and collapsed them by gene and by homoeologous group (triad) (Ramírez‐González *et al*., 2018). These mutations were given equal weight and include stop codon gained, start codon lost, splice donor and acceptor variants and non‐synonymous mutation with a SIFT (sorting intolerance from tolerance) score <0.05 (Kumar *et al*., 2009). We then performed gene and triad‐based burden tests using a single‐locus linear model (CMLM) implemented in GAPIT (genome association and prediction integrated tool) (Lipka *et al*., 2012).

**Figure 1:**
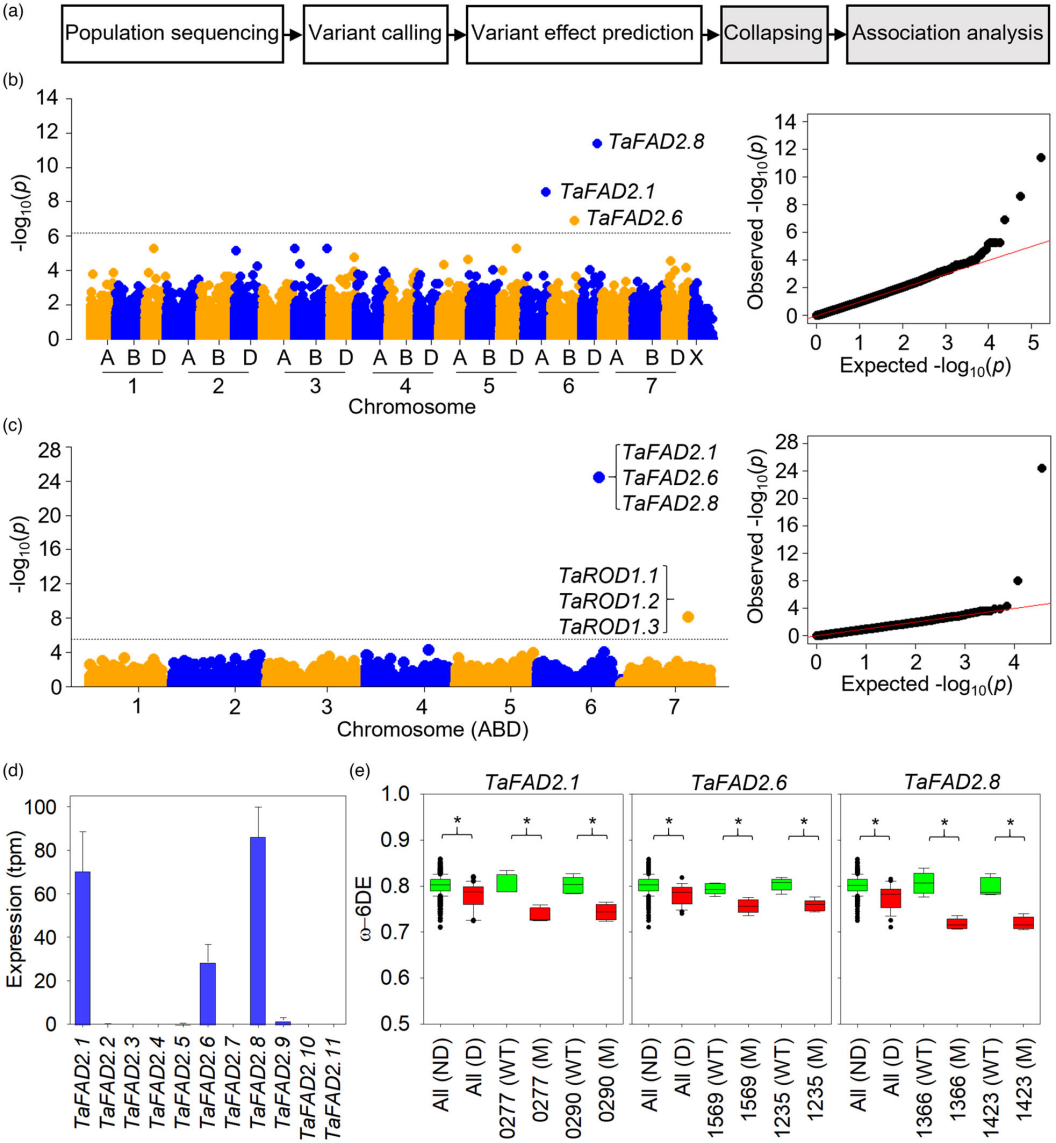
Applying burden tests to the Cadenza exome‐sequenced EMS population to identify genes that control grain ω‐6 fatty acid desaturation efficiency (ω‐6DE). (a) Workflow diagram. White boxes show resources created by Krasileva *et al*. (2017). Manhattan plots showing trait associations with (b) 82 950 genes and (c) 17 616 triads. Collapsed variant frequency threshold = 0.002. Dotted line marks significance threshold after Bonferroni correction for α = 0.05. Putative *TaFAD2* and *TaROD1* genes are highlighted. Quantile–quantile plots shown on right. (d) *TaFAD2* expression in grains at hard dough stage (mean ± SE, *n* = 3). tpm is transcripts per kilobase million. RNA‐seq data from Ramírez‐González *et al*. (2018). (e) Box plots for ω‐6DE in M_4_ grain from all mutant lines containing putative deleterious (D) and non‐deleterious (ND) variants in each *TaFAD2* gene (*n* = 22–1166) and from two independent BC_1_F_2_ homozygous mutants (M) and their wild type segregants (WT) (*n* = 5). Asterisks denote significant differences (*P* < 0.05, unpaired Student's *t*‐test). Cadenza line numbers and *TaFAD2* mutations leading to amino acid substitutions or premature stop codons* are 0277 (W107*), 0290 (P31S), 1569 (W107*), 1235 (L347F), 1366 (Q167*) and 1423 (W92*). [Colour figure can be viewed at wileyonlinelibrary.com]

We identified three genes and two triads that are significantly (*P* < 0.05) associated with ω‐6DE, after applying Bonferroni correction (Figures 1b,c and S1). The three genes *TraesCS6A02G280000*, *TraesCS6B02G309400* and *TraesCS6D02G260200* form one triad and are predicted to encode homologues of FATTY ACID DESATURASE 2 (FAD2) (Hajiahmadi *et al*., 2020). FAD2 is a microsomal ω‐6 fatty acid desaturase that is known to control ω‐6DE in *Arabidopsis thaliana* seeds (Menard *et al*., 2017; Okuley *et al*., 1994). Hexaploid wheat contains eleven putative *FAD2* genes (Hajiahmadi *et al*., 2020), and *TraesCS6A02G280000* (*TaFAD2.1*), *TraesCS6B02G309400* (*TaFAD2.6*) and *TraesCS6D02G260200* (*TaFAD2.8*) are the most strongly expressed in developing grains of cv. Azhurnava (Figure 1d; Ramírez‐González *et al*., 2018). The second triad (TraesCS7A02G378300, TraesCS7B02G280100 and TraesCS7D02G375100) encode putative homologues of REDUCED OLEATE DESATURATION 1 (ROD1), which supplies FAD2 with substrate (Lu *et al*., 2009).

*TaFAD2* and *TaROD1* transcripts are average length for wheat (~1.6 and ~1.5 kb), encoding proteins of ~390 and ~ 300 amino acid residues, respectively. The 1188 M_4_ lines that we screened contained 22–24 putative deleterious mutations in each *TaFAD2* gene, and 6–9 in each *TaROD1* gene, when the M_2_ generation was exome sequenced (Krasileva *et al*., 2017). To confirm that disruption of the *TaFAD2* genes causes a reduction in ω‐6DE, we selected two independent lines with mutations in each gene that had low ω‐6DE in our screen (Figure 1e). We backcrossed them to wildtype and identified five homozygous and five wildtype segregant BC_1_F_2_ plants using KASP (kompetitive allele specific PCR) assays and further confirmed their genotype by DNA sequencing (Krasileva *et al*., 2017). We then analysed the fatty acid composition of their BC_1_F_3_ grains and found that ω‐6DE is significantly (*P* < 0.05) lower in all the homozygous *TaFAD2* mutants (M) versus wildtype (WT) segregants (Figure 1e). The decrease in ω‐6DE is small (<9%), but owing to the high broad‐sense heritability of the trait (H^2^ ~0.9), the effect size is very large (Cohen's *d* > 0.8).

In conclusion, we show that gene and homoeologous group‐based burden tests can identify causal genes for a simple metabolic trait in an exome‐sequenced polyploid mutant population. Many rare‐variant association analysis methods have been developed and may be applicable, including burden tests with more sophisticated weighting, variance‐component and combined tests (Lee *et al*., 2014). We have collapsed point mutations in the Cadenza population, but deletions are also present (Krasileva *et al*., 2017) and could be included. The gene redundancy that exists in polyploid mutant populations likely provides a trade‐off between power and effect size when applying burden tests. Redundancy allows polyploids to tolerate high mutant loads (Krasileva *et al*., 2017), providing smaller populations with more collapsible variants per gene (and homoeologous group). However, redundancy also hides the phenotypic effects of variants (Krasileva *et al*., 2017). It is intuitive that more heritable traits that are controlled by fewer (and larger) genes will likely be more amenable to genetic dissection using burden tests. Mutant populations of tetraploid wheat (Krasileva *et al*., 2017) and many other polyploid crops such oilseed rape (*Brassica napus*) and false flax (*Camelina sativa*) might also be amenable to burden tests.

## Conflict of interest

The authors declare no conflicts of interest.

## Author contributions

P.J.E. conceived the idea and wrote the manuscript. G.M. and P.J.E. conducted the experiments.

## Supporting information


**Figure S1** Complete repeat of the burden test experiment shown in Figure 1b,c.
