# Supplementary material for: Burden tests can be used to map causal genes for a simple metabolic trait in an exome‐sequenced polyploid mutant population
Source: Plant Biotechnol J. 2022 Jul 25;20(10):1850–2. doi: 10.1111/pbi.13890 (PMC9491453; doi:10.1111/pbi.13890)
Supplement: Supplementary file 1 — Figure S1 Complete repeat of the burden test experiment shown in Figure 1b,c. [file PBI-20-1850-s001.docx]

**Supporting materials**

Burden tests can be used to map causal genes for a simple metabolic trait in an exome-sequenced polyploid mutant population

Guillaume N. Menard, Peter J. Eastmond*

Plant Sciences and the Bioeconomy, Rothamsted Research, Harpenden, Hertfordshire, AL5 2JQ, UK *Correspondence: Peter J. Eastmond ([peter.eastmond@rothamsted.ac.uk](mailto:peter.eastmond@rothamsted.ac.uk))


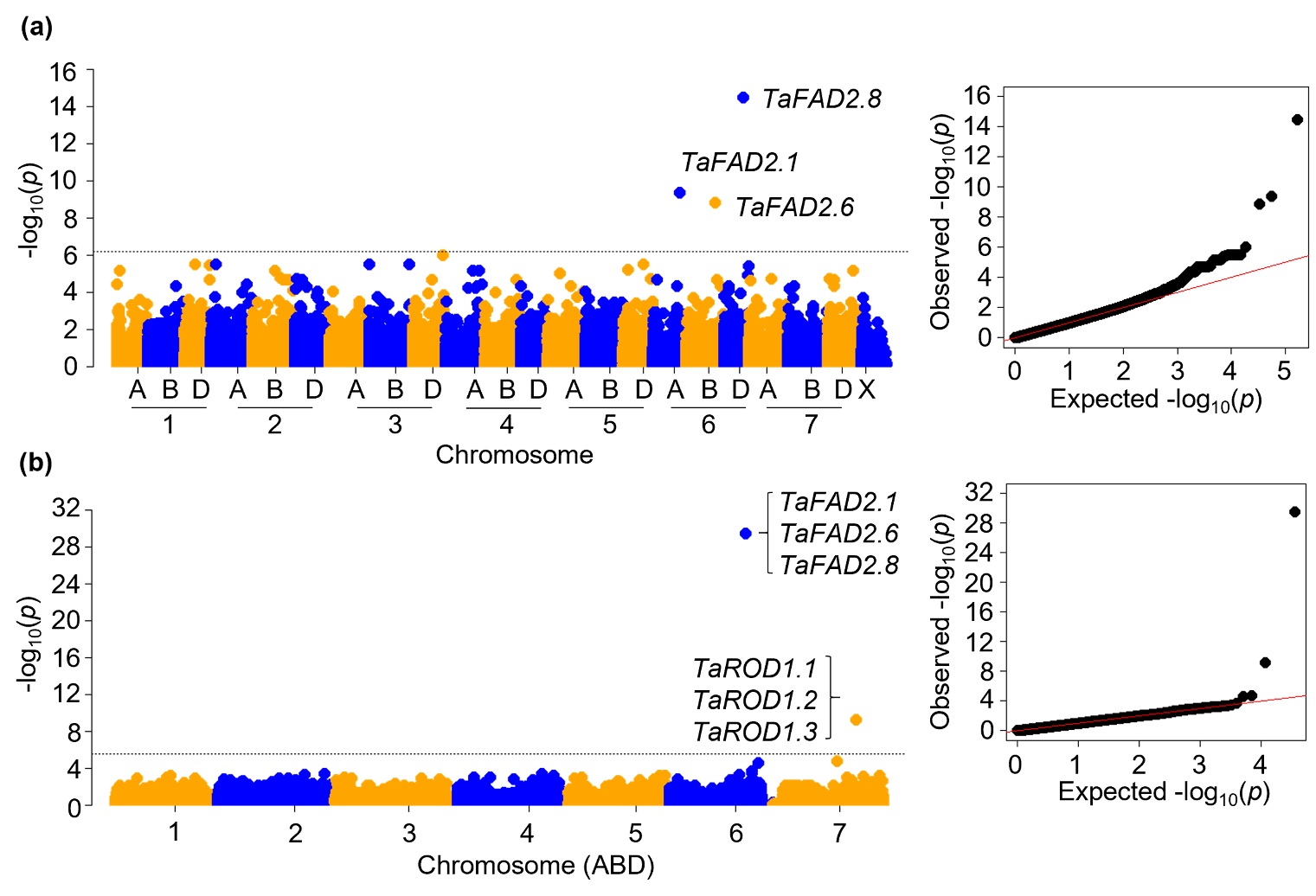


**Figure S1** A complete repeat of the burden test experiment. The fatty acid composition of lipids in individual M_4_ grains from 1188 Cadenza lines was measured and ω-6DE calculated (Menard et al., 2017). Manhattan plots showing trait association with (a) 82,950 genes and (b) 17,616 triads. Collapsed variant frequency threshold = 0.002. Dotted line marks significance threshold after Bonferroni correction for α = 0.05. *FAD2* genes *TaFAD2.1*, *TaFAD2.6* and *TaFAD2.8* are highlighted. Quantile-quantile plots shown on right.
